# Comparable clinical outcomes between octogenarians and young patients following robotic‐assisted unicompartmental knee arthroplasty

**DOI:** 10.1002/jeo2.70491

**Published:** 2025-10-28

**Authors:** Matteo Innocenti, Filippo Leggieri, Mattia Chirico, Alessandro Civinini, Fabrizio Matassi, Roberto Civinini

**Affiliations:** ^1^ Department of Clinical Orthopaedic University of Florence Florence Italy

**Keywords:** age‐related outcomes, clinical outcomes, complications rates, MCID, octogenarians, robotic‐assisted UKA, survival rates, unicompartmental knee arthroplasty

## Abstract

**Purpose:**

Medial unicompartmental knee arthroplasty (mUKA) may offer significant advantages for octogenarian patients due to its reduced surgical burden. The purpose of this study was to analyze the clinical and radiological outcomes, including complications and implant survivorship, in octogenarians following robotic‐assisted mUKA compared to patients aged ≤60 years.

**Methods:**

We conducted a retrospective analysis of prospectively collected data on robotic‐assisted mUKA performed between 2018 and 2022, with a minimum follow‐up of 24 months. Inclusion criteria were patients aged ≥80 years and patients aged ≤60 years. Participants without consent were excluded. A total of 203 patients were included in the final analysis. Propensity score matching was performed to balance the two cohorts (Young: ≤60 years; Octogenarians: ≥80 years) based on body mass index (BMI), Oxford Knee Score (OKS) and Knee Society Score (KSS). The Mann–Whitney *U* test was used to compare continuous variables both preoperatively and postoperatively, as well as clinical improvements across cohorts. The Wilcoxon Signed‐Rank Test and the minimally clinically important difference (MCID) were used to evaluate postoperative clinical outcome improvements within each cohort. The *Chi‐Square* test was used to compare survival and complication rates between groups.

**Results:**

No differences were found between groups in any clinical or radiographic parameters, either preoperatively or postoperatively. Both groups showed comparable and clinically meaningful improvements in KSS and OKS scores. In the octogenarian group, there were two cases of intracortical lateral tibial fractures managed conservatively. One case of subpopliteal deep vein thrombosis (DVT) occurred in the younger cohort. No differences in implant survival were observed between the groups.

**Conclusion:**

Advanced age alone should not preclude octogenarian patients from undergoing robotic‐assisted mUKA, as they can achieve meaningful functional improvements comparable to those of younger patients.

**Level of Evidence:**

Level III.

**Abbreviations**
BMI: body mass index
CI: confidence interval
CT: computed tomography
DVT: deep vein thrombosis
eCDF: empirical cumulative distribution function
HKA: hip–knee–ankle angle
HL: Hodges‐Lehmann
IQR: interquartile range
KSS: Knee Society Score
LMWH: low molecular weight heparin
MAKO: robotic‐assisted system brand name (Stryker)
MCID: minimally clinically important difference
mLDFA: mechanical lateral distal femur angle
MPTA: medial proximal tibia angle
mUKA: medial unicompartmental knee arthroplasty
OKS: Oxford Knee Score
PJI: periprosthetic joint infection
PROMs: patient‐reported outcome measures
PS: Propensity Score
SMD: standardized mean differences
TKA: total knee arthroplasty
UKA: unicompartmental knee arthroplasty

## INTRODUCTION

Octogenarian patients face higher complication rates with total knee arthroplasty (TKA) due to increased comorbidities and poor bone quality compared to the average population [19]. For isolated medial compartment osteoarthritis, medial unicompartmental knee arthroplasty (mUKA) provides reduced surgical burden, shorter operative times, fewer complications, decreased hospital stays and less demanding rehabilitation—critical benefits for frail octogenarians where minimizing surgical disability is essential [6, 10, 18, 24].

While concerns exist regarding UKA survivorship among octogenarians due to the osteoporotic bone, the smaller implant footprint, and the higher risk of tibial subsidence [10, 21, 22], these can be mitigated through precise surgical technique with the correct alignment and implant positioning, while avoiding excessive joint line lowering in varus knees [5]. Additionally, robotic‐assisted technology may help to obtain these parameters while ensuring optimal bone conservation [12, 13, 25].

While similar results have been reported between octogenarian and nonoctogenarian patients undergoing traditional UKA [1, 6, 11, 16], there remains a gap in the literature regarding patient‐reported outcome measures (PROMs) of robotic‐assisted mUKA in elderly patients compared to their younger counterparts aged 60 years or less, despite the well‐established age‐related differences in surgical risk profiles.

Given the favourable outcomes demonstrated in both octogenarian and younger populations, understanding whether elderly patients can achieve comparable functional results to younger patients would provide crucial evidence for establishing mUKA as a preferred medium‐term treatment option in this population.

The purpose of this study was to retrospectively analyze the PROMs, perioperative complications and implant survivorship in octogenarians who underwent robotic‐assisted mUKA compared to a younger population with 60 years of age or less with more than 2 years follow‐up.

## MATERIALS AND METHODS

### Study design and cohorts

This study presents a retrospective analysis of prospectively collected data from 408 consecutive knees undergoing mUKA performed at a single Institution between September 2018 and December 2022 with follow‐up ≥24 months.

The inclusion criteria were age‐based: patients aged ≥80 years old (octogenarians), patients ≤60 years old, and those with at least 2 years of follow‐up. All consecutive cases meeting the age criteria during the study period were included, regardless of patient comorbidity profile or other clinical factors.

**Figure 1:**
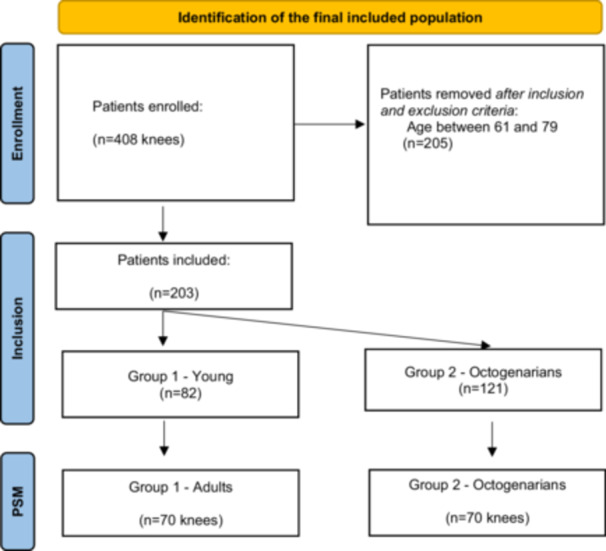
Flowchart of the included studies.

After screening 408 knees in our database against the inclusion and exclusion criteria, a total of 203 were deemed eligible for the analysis in the study (Figure 1).

Propensity score (PS) matching was performed to balance the two cohorts (Young ≤60 years old—Cohort 1; Octogenarians ≥80 years old—Cohort 2) based on body mass index (BMI) and preoperative patient‐reported outcome measures (PROMs). The matching strategy was designed to ensure comparable baseline functional status between groups, as differing preoperative PROMs would have confounded the interpretation of postoperative improvements and would have made drawing definitive conclusions about age‐related differences in outcomes difficult. By achieving similar baseline OKS and KSS scores through propensity score matching, an accurate assessment on whether octogenarians achieved comparable improvements to younger patients was possible, by isolating the effect of age on treatment response while maintaining the natural comorbidity burden that characterizes each age group in real‐world clinical practice. A median age of 58 years (IQR, 55–59) among Cohort 1 and a median of 84 years, (IQR 82–86) among Cohort 2 were found. The complete patient selection process and final cohort distribution are detailed in Figure 1.

### Surgical technique

Preoperative imaging consisted of the standard weight‐bearing long leg X‐rays and the CT MAKOPlasty protocol processed using STRYKER′s platform (Stryker) to generate each individual virtual knee model for preoperative planning.

The surgical procedures were performed with patients positioned supine using the MAKO® robotic‐assisted system (Stryker®) by three highly trained adult knee reconstruction surgeon. Following tourniquet inflation and prophylactic antibiotic administration, a medial parapatellar approach was used routinely.

The same fixed‐bearing metal‐backed cemented UKA implant was used (Restoris MCK partial knee; Stryker). The preferred alignment, implant size and positioning strategies were personalised for each patient with the intention to closely reconstruct the native prearthritic joint surface anatomy with the implants [8]. All the surgical procedures were performed through a midline skin incision with a medial parapatellar arthrotomy with the tourniquet inflated for the entire procedure. Both groups followed standard postoperative care protocols, including pain management and rehabilitation programmes, according to institutional guidelines. All patients followed a standardized DVT prophylaxis protocol that included early mobilization after surgery and daily LMWH injections. All the patients were requiring staying a minimum of 2 days in the hospital following surgery according to the National regulatory policies. No a priori age‐related modifications were made to physical therapy protocols or discharge criteria.

### Data collection

Data such as age, body mass index (BMI), hip–knee–ankle angle (HKA), mechanical lateral distal femur angle (mLDFA) and medial proximal tibia angle (MPTA) were recorded preoperatively and postoperatively [20]. HKA, mLDFA and MPTA were calculated on coronal and lateral knee and full‐length‐leg weight‐bearing X‐rays each time by a single experienced orthopaedic surgeon using TraumaCad® software (Brainlab). The Oxford Knee Score (OKS) and the Knee Society Score (KSS) were preoperatively assessed as well as at 2 years follow‐up [9, 17]. Any intraoperative and postoperative complication was registered. Complications were defined according to The Knee Society's standardized list and definitions of complications of TKAs [7]. Radiographic complications included any modification measurable in the X‐rays including subsidence, osteolysis, radiolucencies, or implant mobilization. Data were collected and measured by two independent Orthopaedic residents.

### Data analysis

Statistical analyses were performed using R software. Descriptive statistics, including median and interquartile range (IQR) were used for continuous. A 1:1 nearest‐neighbour matching algorithm was applied for PS matching using a caliper width of 0.2, based on a binary logistic regression model that included preoperative OKS, KSS and BMI as covariates. Standardized mean differences (SMD) and variance ratios quantified the effectiveness of Ps matching. balance of covariates between groups before and after matching.

Mann–Whitney U test was used to compare radiographic and clinical outcomes both preoperatively and postoperatively, and to compare clinical improvements across the cohorts. Wilcoxon Signed‐Rank Test was used to evaluate the clinical outcome improvements within the cohorts postoperatively. The Minimally Clinically Important Difference (MCID) was calculated using the standard deviation approach. *Chi‐Square* test was used to compare survival between groups and to compare complication rates.

### Power analysis

A post hoc power analysis was conducted with 70 patients per group after propensity matching. Using the observed postoperative scores (Young: KSS 190 ± 26.7, OKS 44.5 ± 9.4; Octogenarian: KSS 194 ± 22.2, OKS 44.0 ± 7.2), the study had low power to detect the small observed differences between groups (16.0% power for 4‐point KSS difference, Cohen's *d* = 0.163; 5.4% power for 0.5‐point OKS difference, Cohen's *d* = 0.060). For detecting clinically meaningful differences at MCID thresholds, the study had moderate power for OKS (64.5% to detect 3.3‐point difference) and lower power for KSS (44.9% to detect 7.6‐point difference). However, since both observed differences (4 points for KSS, 0.5 points for OKS) fell substantially below their respective MCID thresholds (7.6 points for KSS, 3.3 points for OKS), the analysis confirmed true clinical equivalence between age groups rather than statistical underpowering.

## RESULTS

Overall, a final group of 203 knees with a mean follow‐up of 36.3 months (range 24–76 months) were included and 1:1 propensity score matched, resulting in 140 knees divided into two equal cohorts (Figure 1). Covariate distributions before and after propensity score matching (Table 1) demonstrated improved balance of baseline characteristics between young and octogenarian groups, as shown by reduced standardized mean differences and variance ratios for preoperative BMI, KSS and OKS scores (Table 2).

**Table 1:** Baseline covariates distribution before and after PS matching across the cohorts.

	Before propensity score matching					After propensity score matching				
	Young		Octogenarian			Young		Octogenarian		
Variable	Median	IQR	Median	IQR	*p* value	Median	IQR	Median	IQR	*p* value
OKS	28	24.2–35.8	26	23–31	0.021	27	22.2–33	27.5	23–32	0.26
KSS	111	100–121	104	95–116	0.061	108.5	98–120.8	108	98–119.8	0.27
BMI	28	26–31	26	24–30	0.048	28	25.2–30	26.5	24.2–29	0.30

Abbreviations: BMI, body mass index; IQR, interquartile range; KSS, Knee Society Score; OKS, Oxford Knee Score; PS, Propensity score.

**Table 2:** Balance of covariates before and after propensity score matching between young and octogenarian patients.

Balance before PS matching					Balance after PS matching			
Variable	Means treated	Means control	Std. Mean Diff.	Var. ratio	Means treated	Means control	Std. Mean Diff.	Var. ratio
Distance	0.6	0.5	0.5	0.6	0.5	0.5	0.1	1.08
BMI	27.3	28.2	−0.1	1.1	27.4	27.9	−0.1	1.03
KSS	106.1	109.6	−0.1	1.7	109.7	108.6	0.06	1.4
OKS	26.5	29.1	−0.4	0.7	27.3	28.3	−0.1	0.8

Abbreviations: BMI, body mass index; KSS, Knee Society Score; OKS, Oxford Knee Score; PS, Propensity score.

No significant differences were found between young and octogenarian groups in any clinical or radiographic parameters, both preoperatively and postoperatively (Table 3).

**Table 3:** Difference in demographic, clinical and radiographical results after PS matching.

	Young		Octogenarian			
Variable	Median	IQR	Median	IQR	Median difference	*p* value
BMI	28	25.2−30	26.5	24.2−29	−1.5	0.30
KSS	108.5	98−120.8	108	98−119.8	−0.5	0.30
OKS	27	22.2−33	27.5	23−32	0.5	0.62
HKA	176	170−179	174	171−176	−2	0.17
LDFA	88	86−89	89	87−90	1	0.03
MPTA	87	85−89	87	85−88	0	0.34
Postoperative						
KSS	190	169.8−198	194	181.2−200	4	0.08
OKS	44.5	39−48	44	40−46	−0.5	0.12
HKA	178	175−180	178	176−180	0	0.19
LDFA	89	88−90	89	88−90	0	0.27
MPTA	88	84−88	87	85−88	−1	0.11

Abbreviations: BMI, body mass index; HKA, hip–knee–ankle angle; IQR, interquartile range; KSS, Knee Society Score; LDFA, lateral distal femur angle; MPTA, medial proximal tibia angle; OKS, Oxford Knee Score; PS, Propensity score.

**Figure 2:**
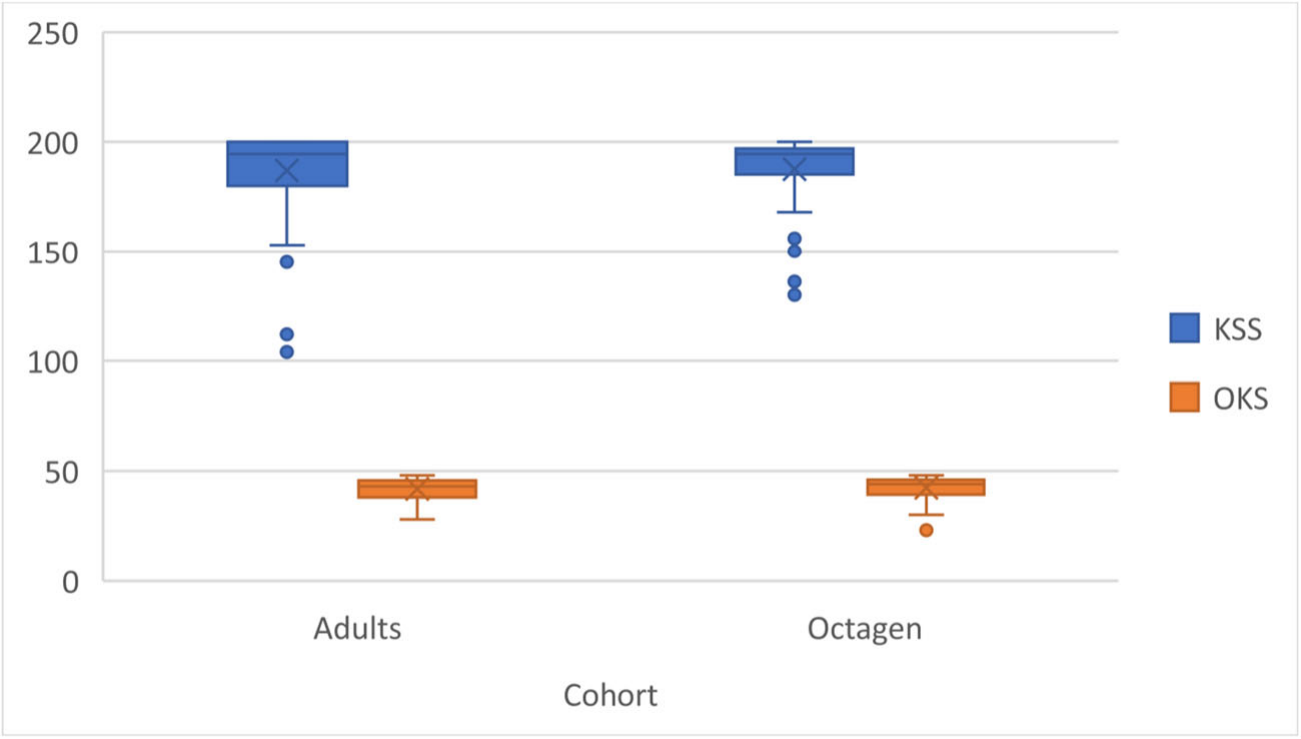
Postoperative functional outcomes across the Cohorts. KSS, Knee Society Score; OKS, Oxford Knee Score.

Both groups showed comparable and clinically meaningful improvements in KSS (young: 76.5 points, octogenarian: 82 points) and OKS scores (young: 15 points, octogenarian: 15 points), with similarly large effect sizes across all measurements (range: 0.83‐0.87) (Table 4). Both young and octogenarian groups exceeded their respective MCID thresholds for KSS (6.92 and 8.39 points) and OKS (3.48 and 3.16 points), with mean improvements substantially higher than these thresholds in both scores. The proportion of patients reaching MCID was remarkably high in both groups, ranging from 89% to 100%, with comparable effect sizes between cohorts (Table 5). Differences in KSS (*p* = 0.0987, HL = −5, 95% CI = −12 to 1) and OKS (*p* = 0.9850, HL = 0, 95% CI = −3 to 3) improvements between the cohorts were not meaningful, with effect sizes of *r* = 0.14 (95% CI = −0.34–0.03) and *r* = 0.00 (95% CI = −0.19 to 0.19), respectively (Figure 2).

**Table 4:** Postoperative clinical improvements across the cohorts.

			Median difference			Effect size		
				95% CI			95% CI	
		*p* value	Value	Low	Upp	Value	Low	Upp
Young	KSS	<0.001	76.5	12.3	105.2	0.8	0.8	0.9
	OKS	<0.001	15	5.2	29	0.8	0.7	0.9
Octo	KSS	<0.001	82	30	106.5	0.8	0.9	1
	OKS	<0.001	14	4	25.7	0.8	0.7	0.9

Abbreviations: CI, confidence interval; KSS, Knee Society Score; OKS, Oxford Knee Score.

**Table 5:** Minimally clinically importance difference distribution across the cohorts.

		MCID		Mean change		Effect size		
Group	Score	Value	95% CI	Value	SD	Value	95% CI	Reached MCID (%)
Young	KSS	6.9	4.6−9.2	72.1	26.7	5.2	4.2–6.1	0.98
	OKS	3.4	2.3–4.6	14.2	9.4	2.0	1.4–2.6	0.90
Octogen	KSS	8.3	5.5–11.1	78.9	22.2	4.7	3.8–5.6	1
	OKS	3.1	2.1–4.2	14.6	7.2	2.3	1.7–2.9	0.97

Abbreviations: CI, confidence interval; KSS, Knee Society Score; MCID, minimally clinically important difference; OKS, Oxford Knee Score.

No differences in survival (100% vs. 100%) or overall complication rate (*Chi‐squared* = 1.91, *p* = 0.167) were found between the groups. There were 2 cases of intracortical lateral tibial plateau fractures occurring as late complications (after 6 months postoperatively) in octogenarian patients following high‐energy falls. Both fractures were managed conservatively without surgical intervention and did not impact functional recovery or cause implant‐related complications at final follow‐up. One case of subpopliteal DVT occurred in the young cohort.

## DISCUSSION

The most important findings of this study were that octogenarian and younger patients achieved similar clinical improvements, with advanced age proving not to be a burden to successful outcomes or increased complication risk following robotic‐assisted mUKA.

Both age groups experienced substantial functional recovery, with improvements in OKS and KSS exceeding thresholds for clinically meaningful change. Notably, octogenarians showed slightly higher median improvements than their younger counterparts, though these differences were not statistically significant. Both groups maintained excellent implant survival rates with comparable complication profiles, where minor issues were successfully managed conservatively without impacting the final mid‐term outcome.

The data suggested that advanced age alone should not be considered a contraindication for knee replacement surgery, as octogenarians demonstrated comparable improvements across all measured parameters. The high proportion of patients exceeding MCID thresholds in both groups, coupled with substantial effect sizes and narrow confidence intervals, indicated that these improvements represented meaningful clinical benefits and support the reliability of these findings.

The rationale for UKA in octogenarians balanced surgical effectiveness with this population′s unique vulnerabilities. Elderly patients face heightened perioperative risks and rehabilitation challenges, making UKA advantageous through reduced surgical burden and less demanding recovery compared to TKA. However, this must be weighed against UKA's technical demands and the catastrophic consequences of surgical failure in elderly patients who cannot afford revision surgery. Registry data showed that UKA achieved similar survival to TKA when performed by experienced and high‐volume surgeons [14, 15]. Since failed UKA requiring conversion would be more detrimental than primary TKA, the selection relied on equivalent outcomes between procedures and precise execution. Enhanced by robotic assistance, UKA may allow octogenarians to benefit from the less invasive approach while maintaining required safety and effectiveness.

Prior literature has demonstrated that joint line depression of 2 mm or more in cases with lower alignment angles (HKA ≤ 175°) significantly increases the risk of tibial component failure due to the combined effect of increased mechanical load from residual varus alignment and reduced bone quality at the depressed joint line position where bone stock is softer [5]. This relationship is particularly critical in octogenarian patients, who typically present with compromised bone quality and are at higher risk for tibial subsidence [4, 23]. The robotic‐assisted technique may help achieve precise implant positioning according to these principles by restricting the tibial component positioning in the soft cancellous bone that results from excessive joint line lowering [2, 3].

To the best of our knowledge, no studies have yet analyzed the outcomes of *robotic‐assisted* mUKAs in this age group compared to a younger cohort, especially <60 yo. Graham et al. performed a propensity score analysis comparing standard UKA outcomes between 44 octogenarians and 132 younger controls aged 65–74 showing no differences in outcomes or complications between groups [6]. Kavanagh et al. comparable risks of morbidity, readmission, or reoperation between octogenarians versus nonoctogenarians evaluating the hospital stay, the discharge status, and the 30‐day morbidity on 5352 patients from the American College of Surgeons National Surgical Quality Improvement Programme data registry [11]. From the same registry, Moore et al. reported a significantly higher rate of adverse events within 30 days postsurgery in octogenarians when compared with younger patients [16]. Unlike previous studies that did not show significant differences in serious adverse events, this analysis revealed increased rates of minor adverse events such as urinary tract infections and blood transfusions, as well as higher instances of mortality, prolonged hospital stays and readmission rates [16]. Recently, from the PearlDiver Mariner database Acuña et al. reported that 1334 octogenarians undergoing UKA experienced similar rates of surgical complications and no differences in rates of PJI, periprosthetic fractures, or aseptic loosening to 5313 with less 80 years old matched controls at 2‐year follow‐up [1].

Additionally, a limited number of studies have evaluated the differences in outcomes between UKA and TKA in octogenarians [21, 22]. Results showed a significantly lower rate of medical complications in the UKA group compared to the TKA group with comparable functional outcomes and satisfaction rates between the groups despite UKA having shorter surgical times and slightly better function in terms of range of motion [21, 22].

The data suggest that advanced age alone should not be considered a contraindication for knee replacement surgery, as octogenarians demonstrated comparable improvements across all measured parameters in the current study. The high proportion of patients exceeding MCID thresholds in both groups, coupled with the substantial effect sizes, indicates that these improvements in both cohorts represent meaningful clinical benefits rather than merely statistical significance. The narrow confidence intervals observed across all measurements provide robust support for the reliability of these findings, suggesting that knee replacement surgery can be considered a viable and effective intervention, regardless of age.

Interestingly, the finding that octogenarian patients achieved comparable outcomes to younger patients suggests that UKA represents a highly effective surgical intervention with excellent results across age groups. However, this equivalence may also highlight the need to evaluate patients using functional performance measures that are not subject to ceiling effects or recall bias, while maintaining objectivity in assessment. The similar patient‐reported outcome scores between vastly different age cohorts could indicate that traditional PROMs may not adequately capture the nuanced functional differences and recovery patterns that likely exist between octogenarians and younger patients, emphasizing the importance of incorporating more sensitive and objective functional performance assessment tools in future studies.

This study has several limitations. The finding of similar complication rates between age groups should be interpreted with caution given our relatively small sample size of 70 patients per group after propensity score matching. This sample size may have limited statistical power to detect meaningful differences in less common complications or adverse events between cohorts. While the mean follow‐up period of 36.3 months provides valuable mid‐term insights, it may has been insufficient to capture long‐term complications such as aseptic loosening or late periprosthetic fractures that are particularly relevant in the octogenarian population with compromised bone quality. The current follow‐up duration, though reasonable for assessing early functional outcomes, precluded definitive conclusions about long‐term implant survival and the risk of late loosening, which typically manifest beyond 5–10 years postoperatively. Therefore, longer‐term studies are needed to establish the durability of robotic‐assisted mUKA in octogenarians and to determine whether the favourable short‐ to mid‐term outcomes observed in this study are sustained over time. Moreover, this octogenarian cohort likely represented a healthier subset of the general elderly population, as these patients successfully passed anesthesiologic evaluation for surgery. While our propensity score matching strategy was essential for isolating age‐related effects on treatment response by balancing preoperative functional status, the inability to match for comorbidities represented a significant limitation given the inherent differences in health status between octogenarians and younger patients. This lack of comorbidity matching may confound our results, as elderly patients typically present with multiple medical conditions that could influence surgical outcomes, recovery patterns and functional improvements. Additionally, the matching approach based on preoperative PROMs introduced selection bias toward more functionally capable elderly patients who may not represent the typical frail elderly population with multiple comorbidities. Therefore, these results may have been generalizable to all octogenarians with knee arthritis, particularly those with significant comorbidities or functional limitations.

## CONCLUSIONS

Robotic‐assisted mUKA demonstrated comparable clinical improvements and mid‐term outcomes between octogenarians ≥80 years old and younger patients ≤60 years old, suggesting that the reduced surgical burden of this less invasive procedure makes it a feasible option for carefully selected elderly patients.

## AUTHOR CONTRIBUTIONS

Roberto Civinini and Matteo Innocenti contributed to the study conception and design. Material preparation and data collection were performed by all authors. Data analyses were performed by Filippo Leggieri. Mattia Chirico assessed the interpretation of the data results and supervised the study from inception. The first draft of the manuscript was written by Alessandro Civinini and Filippo Leggieri. Roberto Civinini, Matteo Innocenti and Fabrizio Matassi reviewed the draft, and all the authors commented on previous versions of the manuscript. All the authors read and approved the final manuscript.

## CONFLICT OF INTEREST STATEMENT

The authors declare no conflicts of interest.

## ETHICS STATEMENT

The study adhered to the principles outlined in the Declaration of Helsinki. The Local Ethics Committees reviewed the study protocol and determined that no ethical approval was required, given the purely retrospective and observational nature of the design. All patients provided informed consent before enrolment in the study.

## Data Availability

The data sets used and/or analyzed during the current study are available from the corresponding author on request.
